# Caffeic acid and hydroxytyrosol have anti-obesogenic properties in zebrafish and rainbow trout models

**DOI:** 10.1371/journal.pone.0178833

**Published:** 2017-06-01

**Authors:** Esmail Lutfi, Patrick J. Babin, Joaquim Gutiérrez, Encarnación Capilla, Isabel Navarro

**Affiliations:** 1 Department of Cell Biology, Physiology and Immunology, Faculty of Biology, University of Barcelona, Barcelona, Spain; 2 Maladies Rares: Génétique et Métabolisme (MRGM), University of Bordeaux, INSERM, U12211, Pessac, France; Universita degli Studi di Catania, ITALY

## Abstract

Some natural products, known sources of bioactive compounds with a wide range of properties, may have therapeutic values in human health and diseases, as well as agronomic applications. The effect of three compounds of plant origin with well-known dietary antioxidant properties, astaxanthin (ATX), caffeic acid (CA) and hydroxytyrosol (HT), on zebrafish (*Danio rerio*) larval adiposity and rainbow trout (*Onchorynchus mykiss*) adipocytes was assessed. The zebrafish obesogenic test (ZOT) demonstrated the anti-obesogenic activity of CA and HT. These compounds were able to counteract the obesogenic effect produced by the peroxisome proliferator-activated receptor gamma (PPARγ) agonist, rosiglitazone (RGZ). CA and HT suppressed RGZ-increased PPARγ protein expression and lipid accumulation in primary-cultured rainbow trout adipocytes. HT also significantly reduced plasma triacylglycerol concentrations, as well as mRNA levels of the *fasn* adipogenic gene in the adipose tissue of HT-injected rainbow trout. In conclusion, *in vitro* and *in vivo* approaches demonstrated the anti-obesogenic potential of CA and HT on teleost fish models that may be relevant for studying their molecular mode of action. Further studies are required to evaluate the effect of these bioactive components as food supplements for modulating adiposity in farmed fish.

## Introduction

Obesity has become a worldwide epidemic and is considered one of the most serious public health problems of our time [1,2]. Overweight and obesity occur when energy intake exceeds energy expenditure, leading to increased storage of triacylglycerols (TAG), mainly in white adipose tissue (WAT). In addition to TAG storage in adipocyte lipid droplets, WAT has been recognized as a multi-functional endocrine organ that plays a critical role in modulating several physiological processes, such as appetite, whole-body energy metabolism and homeostasis, as well as tissue inflammation responses [3]. Consequently, concomitant with enlarged fat storage, pathological overgrowth of WAT is associated with a range of related problems, including type II diabetes, insulin resistance, hypertension and cardiovascular diseases [4].

One of the key molecules that modulates WAT activity in response to extrinsic signals is peroxisome proliferator-activated receptor gamma (PPARγ), a master regulator of adipogenesis that activates the transcription of a large number of genes involved in adipocyte differentiation and lipid accumulation [5]. Furthermore, PPARγ controls the expression of many factors secreted by WAT that influence insulin sensitivity, which in turn, modulate the expression of genes involved in glucose homeostasis [6]. Impaired PPARγ signaling, expression and/or activation are thus implicated in the prevalence of metabolic obesogenesis and weight-related diseases, such as diabetes. The most widely studied therapeutic use of PPARγ has been in the treatment of insulin resistance and type II diabetes. Synthetic ligands/agonists of PPARγ, e.g. thiazolidinediones, commonly used as insulin sensitizers for treating hyperglycemia in patients with type II diabetes, are of great clinical significance [7]. Nevertheless, despite their effectiveness in normalizing blood glucose levels, these compounds present detrimental side effects, such as weight gain, edema and cardiovascular complications [8]. Thus, the discovery or development of new compounds that modulate the PPARγ signaling pathway more effectively and safely, while promoting health benefits, is currently a matter of great interest.

Throughout history, natural products have provided a rich source of inspiration for drug discovery. Significant research has recently been undertaken to identify PPARγ modulators, with the aim of formulating a novel treatment to maximize antiobesity effects, in addition to antioxidant and protective properties [9]. Natural antioxidants modulate WAT inflammation produced by the overproduction of reactive oxygen species or pathological processes associated with obesity. While caffeic acid (CA), hydroxytyrosol (HT) and astaxanthin (ATX) are interesting examples of dietary compounds with proven antioxidant properties [10–12], their specific potential for treating obesity has not been fully recognized. Furthermore, the increasing use of plant-based aquafeeds has aroused great interest in the identification of new vegetal ingredients that may respond not only to the demand for sustainable aquaculture, but also, to help develop new diets that may reduce unwanted perivisceral WAT in farmed fish.

In basic research, mammalian models (e.g. mice and rats primarily) have been traditionally used in human physiology and disease research, due to their anatomical and physiological similarities [13]. Nevertheless, they could be unsuited for certain types of studies [14]. In the past decade, teleost species have been regarded as excellent alternative models for studying human diseases [15,16] and now constitute an emerging method for assessing bioactive compounds in food research [17]. A number of *in vitro* and *in vivo* studies have highlighted the applicability of several fish species within the areas of lipid metabolism and adipose tissue biology [18–23]. Besides its simplicity and numerous other advantages, fish research models such as zebrafish (*Danio rerio*) or rainbow trout (*Onchorynchus mykiss*), are very promising for obesity research, as most of the metabolic pathways linked to the lipid metabolism are conserved between mammals and teleost fish [24–26]. Indeed, histological studies have revealed also evolutionarily conserved morphological structures of teleost adipocytes [27–29].

In this study, three selected antioxidant dietary compounds (CA, HT, and ATX) were used *in vitro* and *in vivo*, to assess their potential anti-obesogenic effect on zebrafish and rainbow trout models.

## Materials and methods

### Animal care and ethics statement

Wild-type zebrafish were produced in our facilities at the University of Bordeaux in accordance with the French Directive (Ministère de l’Agriculture, de l’Agroalimentaire et de la Forêt), under permit number A33-522-6. All experiments were conducted in conformity with the European Communities Council Directive (2010/63/EU) on the protection of animals used for scientific purposes and local French legislation on the care and use of laboratory animals. Larvae were obtained by natural mating and raised in embryo water (90 μg/ml Instant Ocean [Aquarium Systems, Sarrebourg, France], 0.58 mM CaSO_4_, 2H_2_O, dissolved in reverse-osmosis purified water) at 28.5°C with an 11L:13D photoperiod. From 5 days postfertilization until day 15, larvae were fed *ad libitum* on ZF Biolabs formulated diet flakes (Tres Cantos, Spain). They were then nourished with standard diet (SD) for late larvae (TetraMin Baby, Tetra GmbH, Melle, Germany). Animal stages were recorded according to standard length, i.e. the distance from the rostral tip of the larva to the base of the caudal fin.

Juvenile rainbow trout, body weight approximately 80 g for *in vivo* studies and 250 g for extracting WAT to be used in adipocyte primary cultures, were obtained from the “Viveros de los Pirineos” fish farm (El Grado, Huesca, Spain). Animals were maintained according to the Ethics and Animal Care Committee of the University of Barcelona, following the regulations and procedures established by the Spanish and Catalan governments (CEEA 170/14, CEEA 311/15, DAAM 7952).

### Reagents

HT (ref. 70604, CAS N°10597-60-1) and rosiglitazone (RGZ) (ref. 71740, CAS N°122320-73-4) were purchased from Cayman chemicals (Ann Arbor, MI). CA (ref. C0625, CAS N°331-39-5), sesame oil (ref. S3547), DMSO (ref. D8418), and ethyl 3-aminobenzoate methanesulfonate (MS-222) (ref. E10521) were provided by Sigma-Aldrich (Tres Cantos, Spain). Certified analytical grade ATX (ref. DRE-CA10307000, CAS N°472-61-7) was purchased from Dr. Ehrenstorfer GmbH (Augsburg, Germany). Stock solutions were stored at -20°C and working solutions were diluted in 0.1% DMSO on the day of the experiment.

### Zebrafish obesogenic test (ZOT)

The short-term ZOT assay, using Nile red staining, is a non-invasive *in vivo* method for visualizing the effects of the molecules tested on the adiposity dynamics of zebrafish larvae by fluorescence microscopy. The 3-day, *in vivo*, animal treatment protocol was performed as previously described [30]. In replicated experiments, ten larvae ranging from 7 to 9 mm standard length were used per group. Larvae were exposed to the selected compounds or to vehicle alone for one day in a fasting state. All larvae were kept in a fasting state for 24 h prior to exposure and until the end of the trial, to avoid food auto-fluorescence. Treatments were as follows: vehicle control (CT) (0.1% DMSO), CA (0.1% DMSO plus 50 μM CA), HT (0.1% DMSO plus 100 μM HT), and ATX (0.1% DMSO plus 100 μM ATX). Concentrations of each molecule tested were the highest that did not induce any mortality. Quantitative analysis was performed, as previously described [30], by recording the image area of Nile red green fluorescence as a percentage of initial adiposity, using ImageJ software (National Institutes of Health, Bethesda, MD, USA).

### Rainbow trout adipocyte cell cultures

All cell-culture reagents were purchased from Sigma-Aldrich (Tres Cantos, Spain) and Life Technologies (Alcobendas, Spain). All plastic items and glass cover slips were obtained from Nunc (LabClinics, Barcelona, Spain). Cells were cultured according to the previously established procedure [31], with perivisceral WAT from four to six fish per culture. After counting, cells were seeded at a final density of 2–2.5·10^4^ cells/cm^2^ in 1% gelatin on pretreated six-well plates (9.6 cm^2^/well) for real-time quantitative PCR (qRT-PCR) analyses or twelve-well plates (2.55 cm^2^/well), with or without coverslips, for immunofluorescence or Oil red O (ORO) staining, respectively. Plates were kept at 18°C in Leibovitz's L-15 growth medium, supplemented with 10% fetal bovine serum and 1% antibiotic-antimycotic solution (growth medium, GM). When necessary, the standard procedure used for cell differentiation was the following: once confluence was reached (day 7), cells were induced to differentiate by incubating them with a differentiation medium (DM) based on GM and containing 10 μg/mL insulin, 0.5 mM 3-isobutyl-1-methylxanthine and 0.25 μM dexamethasone. The medium was changed every 2 days throughout the procedure.

### Immunofluorescence assay

Post-confluent pre-adipocyte cells from day 7 of culture were incubated with vehicle CT (DM ± 0.1% DMSO) or vehicle plus CA (50 μM), HT (100 μM) or RGZ (1 μM), or a combination of CA or HT with RGZ, for 24 h. RGZ was used as a potential rainbow trout peroxisome proliferator-activated receptor gamma (PPARγ) agonist. PPARγ was detected by immunofluorescence, using the protocol described by [32]. The polyclonal rabbit anti-PPARγ (H-100) was purchased from Santa Cruz Biotechnology (Santa Cruz, CA). Secondary Alexa Fluor^®^ conjugated antibody (A21069, goat anti-rabbit 568) was purchased from Life Technologies (Alcobendas, Spain). Nuclei were counterstained with Hoechst (H1399, Life Technologies, Alcobendas, Spain). Images were obtained at 36x magnification on a Leica TCS-SP5 confocal microscope. Nucleus fluorescence was quantified and normalized to the total number of nuclei in the same field, using ImageJ software (National Institutes of Health, Bethesda, MD, USA).

### Western blot analysis

Post-confluent pre-adipocyte cells from day 7 of culture were treated as described in the previous immunofluorescence section. Protein extraction and Western blot analysis were performed using the protocol described by [32]. Briefly, the amount of protein from each sample was measured [33] and 20 μg were subjected to electrophoresis (SDS-PAGE) on 10% polyacrylamide gels (125 V for 1 h 30 min). After overnight transfer to a PVDF membrane, a staining with Ponceau S solution (Sigma-Aldrich, Tres Cantos, Spain) was performed, showing similar amounts of transferred proteins on each lane and membranes were scanned for posterior band quantification. Subsequently, membranes were washed and then blocked in non-fat milk 5% and incubated with polyclonal rabbit anti-PPARγ (H-100), an antibody that has been previously shown to successfully cross-react with rainbow trout [31,34]. After washing, membranes were incubated with a peroxidase-conjugated secondary goat anti-rabbit antibody (Cat. No. 31460. Thermo Scientific, Alcobendas, Spain). The immunoreactive band was visualized using an enhanced chemiluminescence kit (Pierce ECL Western blotting Substrate; Thermo Scientific, Alcobendas, Spain) and quantified by densitometric scanning using ImageJ software (National Institutes of Health, Bethesda, MD, USA). Results from the densitometry analysis of each specific band were normalized by the densitometry values of the most abundant band of Ponceau S staining as previously reported [35]. In support of this methodology, it has been shown that reversible Ponceau S staining can be used advantageously over specific proteins detection for quality or control of equal loading in Western blotting [36,37].

### Oil red O staining and lipid quantification

After confluence (day 7), cells were incubated with vehicle CT (DM ± 0.1% DMSO) or vehicle plus CA (50 μM), HT (100 μM), LIP (10 μl/ml) or the indicated combination of CA or HT with LIP, for 72 h. Cell differentiation and lipid accumulation were analyzed by ORO staining, as described elsewhere [38], with minor modifications. Briefly, cells were fixed with 10% formalin for 1 h. Fixed cells were rinsed with PBS, stained with 0.3% ORO prepared in 36% tri-ethyl phosphate for 2 h, and then rinsed three times with distilled water. A 100% 2-propanol solution was used to elute the ORO dye and absorbance was measured at 490 nm. The cells were then stained with Coomassie blue for 1 h and proteins were extracted using 85% propylene glycol at 60°C for 1 h. Lipid quantification was calculated as the absorbance measured at 490 nm divided by the measurement corresponding to protein at 630 nm.

### Cell viability assay

Pre-confluent pre-adipocyte cells (day 5 of culture) were incubated with vehicle CT (GM) or vehicle plus CA (50 μM), HT (100 μM), RGZ (1 μM), or a combination of CA or HT with RGZ, for 24 h. The methylthiazolyldiphenyl-tetrazolium bromide (MTT) assay was performed as previously described elsewhere [38]. Briefly, after 24 h incubation with a final concentration of 0.5 mg/mL MTT, cells were washed with PBS and the blue formazan crystals that formed were resuspended in 250 μL DMSO per well for 2 h. Cell viability values were obtained from the absorbance measured at 570 nm, with 680 nm as the reference wavelength, using a microplate reader (Infinite 200, Tecan).

### Measuring cell proliferation

Pre-confluent pre-adipocyte cells (day 5 of culture) were incubated as described in the previous cell viability section. Cell proliferation was evaluated by immunocytochemical detection of proliferating cell nuclear antigen (PCNA), using a commercial staining kit (Cat. No. 93–1143, Life Technologies, Alcobendas, Spain). In brief, after 24 h incubation (see above), cells were washed and fixed in 4% paraformaldehyde (PFA, Sigma-Aldrich, Spain) at room temperature for 15 min. Subsequently, coverslips were post-fixed in 50% and 70% ethanol for 5 min and incubated in PCNA staining reagents, following the manufacturer’s suggested protocol. The amount of PCNA-labeled nuclei (positive cells) was evaluated and normalized to the number of nuclei in that field, using ImageJ software (National Institutes of Health, Bethesda, MD, USA). Five to ten images were taken per coverslip with a CC2 camera coupled to a microscope at 40x using analySIS (Soft Imaging System) software.

### In vivo experimental treatment in rainbow trout

After 15 days' acclimation in our facilities at 15°C, juvenile rainbow trout fasted for 24 h were then anesthetized with MS-222 (0.1 g/L) prior to receiving an intraperitoneal injection of 4.64 μL volume per g body weight. Treatments were as follows: vehicle CT containing DMSO diluted in sesame oil (1:3, v/v), vehicle plus CA at 10 μg/g body weight, and vehicle plus HT at 20 μg/g body weight. After 24 h exposure to the compounds in a fasting state, trout were anesthetized, sacrificed by a blow to the head, and blood samples were taken from the caudal aorta. Liver and perivisceral WAT samples were then harvested and preserved at −80°C pending analysis. None of the molecules tested induced mortality at the concentrations used.

### Biochemical analysis of plasma parameters

Plasma samples were analyzed using commercial enzyme kits: glucose (Monlab, Barcelona, Spain), non-esterified fatty acids (NEFAs, Wako Chemicals GmbH, Neuss, Germany), and TAG and glycerol (Sigma-Aldrich, Tres Cantos, Spain).

### RNA extraction, cDNA synthesis and qRT-PCR

Total RNAs from WAT (100 mg), liver (50 mg), and primary adipocyte cells (3 wells) were extracted using TriReagent (Ambion, Alcobendas, Spain), according to the manufacturer's recommendations. Treatments for *in vitro* studies were as follows: vehicle CT (DM ± 0.1% DMSO) or vehicle plus CA (50 μM), HT (100 μM), RGZ (1 μM), LIP (10 μl/ml) or the indicated combination of CA or HT with RGZ or LIP, for 24 h. A ND-2000 NanoDrop spectrophotometer (Thermo Fisher Scientific, Alcobendas, Spain) was used to quantify isolated RNAs and 500 ng (cell culture) or 1 μg (tissue) samples of total RNAs were treated with DNase I (Life Technologies, Alcobendas, Spain), following the manufacturer’s protocol, to remove all genomic DNA. Afterwards, total RNAs were reverse transcribed with the Transcriptor First Strand cDNA synthesis Kit (Roche, Sant Cugat del Valles, Spain). qRT-PCR was performed as previously described [39]. All the analyses were performed in triplicate using 384-well plates with 2.5 μL itaq SYBR Green Supermix (Bio-Rad, El Prat de Llobregat, Spain), 250 nM forward and reverse primers, and 1 μL cDNA for each sample, in a final volume of 5 μL. The primers were specific for acyl-CoA synthetase-1 (*acsl1*), beta-actin (*actb*), CCAAT/enhancer-binding protein alpha (*cebpa*), elongation factor 1 alpha (*ef1α*), fatty acid synthase (*fasn*), 3-hydroxyacyl-CoA dehydrogenase (*hoad*), hormone sensitive lipase (*lipe*), lipoprotein lipase (*lpl*), adipose triglyceride lipase (*pnpla2*), peroxisome proliferator activated receptor beta (*pparb*) and gamma (*pparg*), and *ubiquitin* (S1 Table).

### Statistical analyses

Data were analyzed using IBM SPSS Statistics v.22 (IBM, Armonk, USA) and GraphPad prism 6 (La Jolla, USA, www.graphpad.com) and presented as mean ± SEM, unless stated otherwise. Normal distribution was first analyzed using the Shapiro-Wilk test, followed by Levene’s to test homogeneity of variances. Statistical significance was assessed by two-tailed unpaired Student’s t-test or one-way analysis of variance (ANOVA), followed by the Tukey *post-hoc* test. When data did not follow a normal distribution, a non-parametric Kruskal-Wallis test was applied, followed by a paired U-Mann Whitney test. Statistical differences were considered significant for all analyses when *p*-value ≤ 0.05, indicated by asterisks: *p ≤ 0.05, **p ≤ 0.01, ***p ≤ 0.0001.

The mRNA level of each gene analyzed was calculated relative to the corresponding reference genes, i.e. geometric means of *ef1a* and *ubiquitin* for the *in vitro* cell culture assays and *ef1a* and *actb* for *in vivo* assays, using the Pfaffl method [40] implemented in the Biorad CFX manager 3.1 (Bio-Rad, El Prat de Llobregat, Spain).

## Results

### Identifying compounds of plant origin with antioxidant properties able to decrease zebrafish larval adiposity in vivo

**Fig 1:**
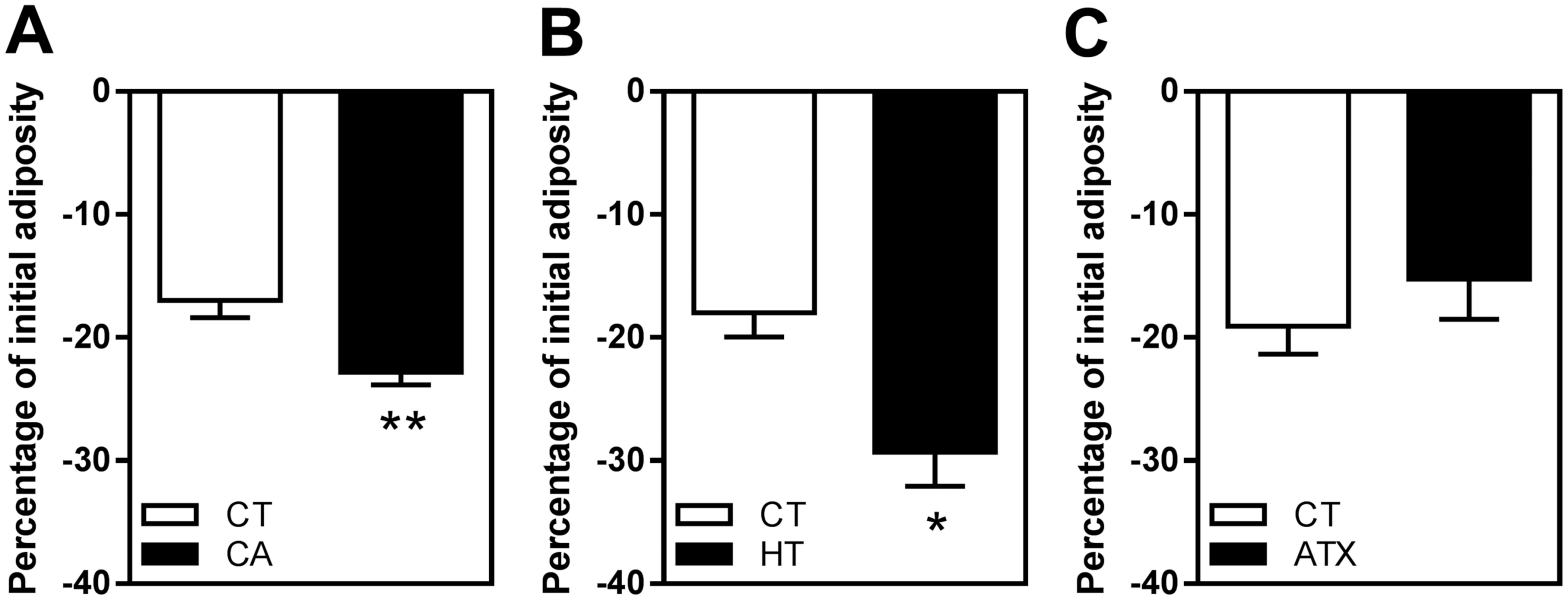
Identification of selected molecules of plant origin able to decrease adiposity *in vivo*. ZOT was conducted on larvae with a standard-length distribution from 7 to 9 mm and initially nourished with SD. Adiposity was quantified in fasting larvae in fish water with 0.1% DMSO as a vehicle control (CT) or 0.1% DMSO plus 50 μM CA (A), 100 μM HT (B), or 100 μM ATX (C). For each larva enrolled, WAT dynamics is expressed as a percentage of initial adiposity. Values are mean ± SEM, n = 4–6 independent experiments (10 animals per group). *P ≤ 0.05, **P ≤ 0.01 compared to CT, using two-tailed unpaired Student's t-test.

To investigate the effect of CA, HT and ATX on the dynamics of zebrafish adiposity, ZOT was used as a non-invasive *in vivo* method for visualizing lipid droplets in living larvae by fluorescence microscopy [30]. The whole-body adiposity dynamics of each larva was expressed as a percentage of variation in Nile red fluorescence signal areas in fish previously fed on standard diet (SD) after one-day's exposure in a fasting state. The results revealed that exposure to 50 μM CA significantly decreased larval adiposity compared to CT-exposed fish (−22.83 ± 1.02% in CA *versus* −16.99 ± 1.29% with CT, *p* ≤ 0.01) (Fig 1A). The same effect was observed with 100 μM HT (−29.72 ± 2.74% in HT *versus* −17.98 ± 1.97% with CT, *p* ≤ 0.05) (Fig 1B). In contrast, there was no change in adiposity following exposure to 100 μM ATX (Fig 1C).

### Differential effect of CA and HT on WAT dynamics in different body parts

**Fig 2:**
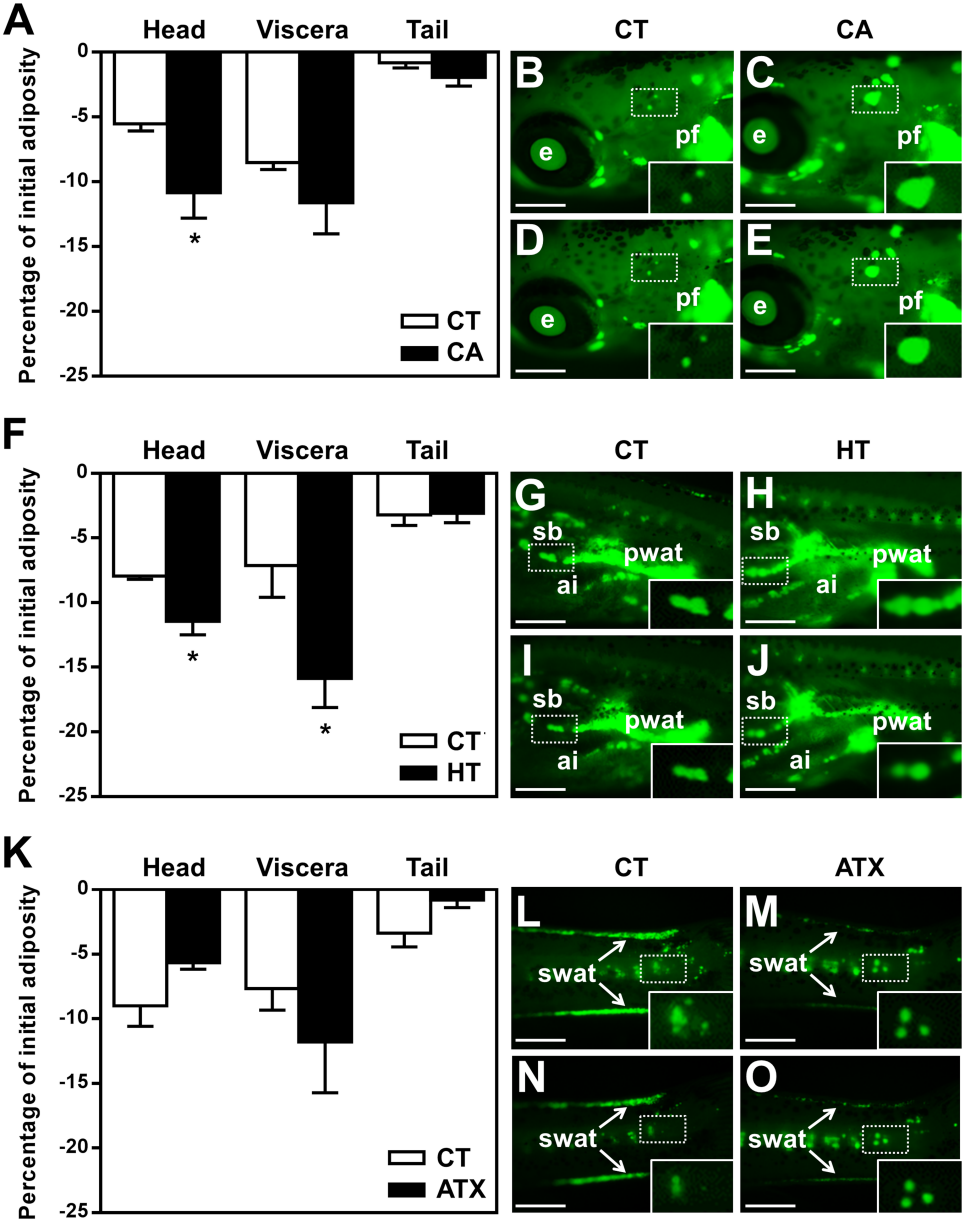
Differential effect of selected vegetal molecules of plant origin on WAT dynamics in different body parts of zebrafish larvae. Quantitative analysis of WAT dynamics was performed according to the ZOT protocol and the results are expressed as a percentage of initial adiposity relative to the amount of WAT fluorescence signal attached to each body region. Fasting larvae were exposed to 50 μM CA in 0.1% DMSO (A, C, E), 100 μM HT in 0.1% DMSO (F, H, J), 100 μM ATX in 0.1% DMSO (K, M, O), or 0.1% DMSO as a vehicle CT (A, B, D, F, G, I, K, L, N). Images of relevant regions in representative larvae are presented: head (B-E), viscera (G-J), and tail (L-O). Lateral views, anterior part on the left and dorsal part at the top, under fluorescence microscope after Nile red staining, recorded before (B, C, G, H, L, M) and after 24 h treatment (D, E, I, J, N, O) with (CA, HT, ATX) or without (CT) exposure to the compounds. Insets in each image are enlarged views of isolated adipocytes or groups of adipocytes from each panel, marked by a white rectangle. Values are mean + SEM, n = 4–6 independent experiments (10 animals per group). *p ≤ 0.05 compared to control for each region, using Student's t-test. Scale bar, 500 μm. Abbreviations: e, eye; pf, pectoral fin; pwat, perivisceral white adipose tissue; sb, swim bladder; ai, anterior intestine; swat, subcutaneous white adipose tissue.

ZOT was used to study WAT dynamics in different body parts of zebrafish larvae, during exposure to the selected bioactive compounds. Perivisceral WAT accounted for around 60% of adiposity at these zebrafish larval developmental stages (S1 Fig). The remaining WAT in the head and tail regions represented around 30% and 10%, respectively (S1 Fig). Results showed that exposure to CA mainly affected adiposity in the head region, compared to the same region in CT fish (−10.85 ± 1.95% in CA *versus* −5.53 ± 0.54% with CT, *p* ≤ 0.05) (Fig 2A). HT also led to a significant decrease in adiposity in the head (−11.46 ± 1.03% in HT *versus* −7.95 ± 0.24% with CT, *p* ≤ 0.05) and viscera (−15.87 ± 2.23% in HT *versus* −7.13 ± 2.46% with CT, *p* ≤ 0.05) compared to CT (Fig 2F). In contrast, ATX did not have a significant anti-obesogenic effect in any of the body regions (Fig 2K). Fluorescent images of isolated or grouped adipocytes revealed a marked decrease in oil droplet size following treatment with CA or HT (Fig 2C, 2E, 2H and 2J).

### Potential antagonist effect of CA and HT on PPARγ signaling pathway

**Fig 3:**
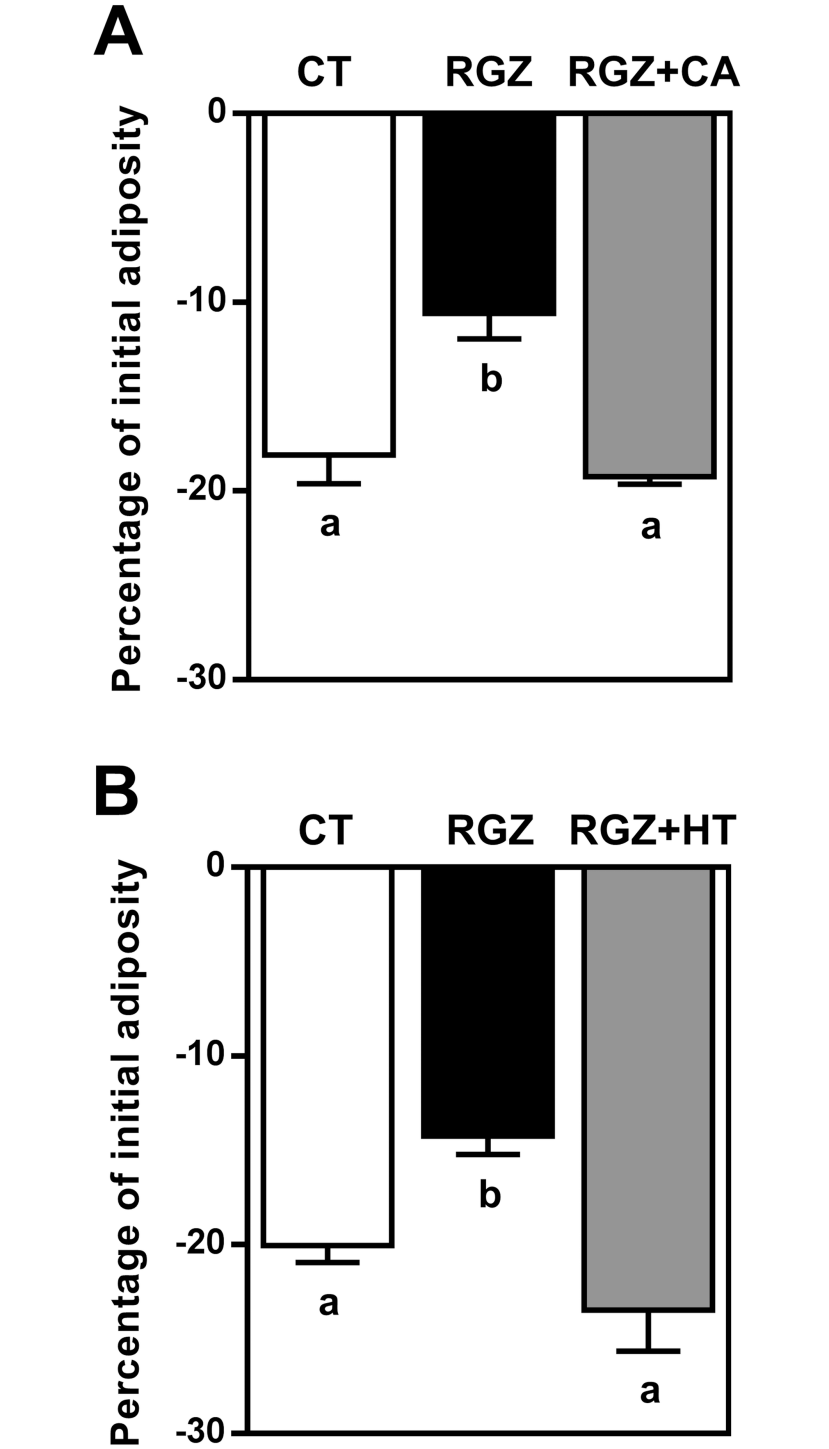
CA and HT abolished the *in vivo* obesogenic effect of RGZ. ZOT was applied to larvae previously nourished with SD. Adiposity was quantified in the presence of 0.1% DMSO as a vehicle CT or 0.1% DMSO plus the indicated combination of compounds. Exposure to 1 μM RGZ induced a significantly smaller decrease in adiposity compared with CT. The effect of RGZ was abolished by CA 50 μM (A) and HT (100 μM) (B). Values are mean ± SEM, n = 4 independent experiments (10 animals per group). Significant differences are shown as different letters (*p* ≤ 0.05) using one-way ANOVA test followed by Tukey’s *post hoc* test.

In view of the significant decrease in zebrafish larvae adiposity observed after treatment with CA and HT, the next step was to elucidate whether these compounds were capable of counteracting the obesogenic effect of RGZ, a potential PPARγ agonist, *in vivo*. RGZ at 1 μM on an SD background diet was shown to prevent adiposity loss in fasting condition: −10.59 ± 1.35% in RGZ *versus* −18.1 ± 1.52% with CT, *p* ≤ 0.01 (Fig 3A) and −14.23 ± 0.98% in RGZ *versus* −20.04 ± 0.88% with CT, *p* ≤ 0.05 (Fig 3B). Simultaneous exposure of the larvae to RGZ and CA revealed that the obesogenic effect of RGZ was abolished by this compound (−19.24 ± 0.40%) (Fig 3A). A similar effect was obtained following co-incubation with RGZ and HT (−23.47 ± 2.17%) (Fig 3B).

### Characterization of CA and HT effects on primary-cultured rainbow trout adipocytes

**Fig 4:**
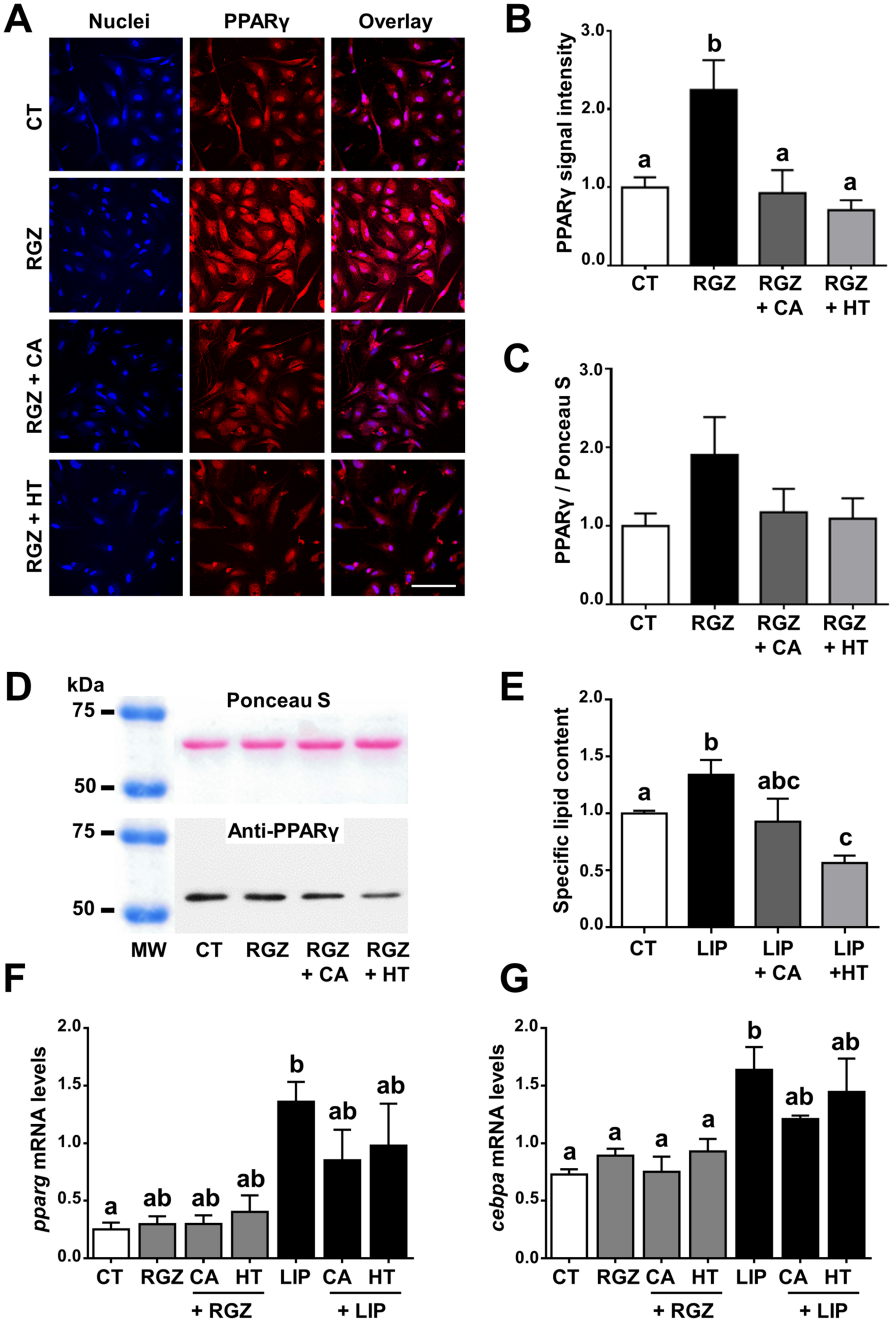
Characterization of potential PPARγ signaling pathway antagonism and inhibition of adipogenesis produced by CA and HT in primary-cultured rainbow trout adipocytes. Representative PPARγ immunofluorescence images (A), quantification of PPARγ immunofluorescence protein signal (B), anti-PPARγ immunoreactive band and quantification of PPARγ protein expression by Western blot (C, D), specific lipid content (E) and mRNA levels of adipogenic genes *pparg* (F) and *cebpa* (G). Immunofluorescence images show Hoechst nuclei staining (left panels), PPARγ (medium panels) and overlay (right panels). Scale bar, 100 μm. For both protein expression analyses (immunofluorescence, A and B; Western blot, C and D) cells were incubated with vehicle (DM) plus CA (50 μM), HT (100 μM), RGZ (1 μM), or the indicated combination of CA or HT with RGZ, or vehicle CT alone, for 24 h (day 7 of culture). RGZ was used as a potential rainbow trout PPARγ agonist. Representative Western blot images of anti-PPARγ immunoreactive band (top) and the same membrane labelled with Ponceau S (bottom) (D). Lipid content expressed spectrophotometrically as the ratio of absorbance value between ORO and Coomassie blue staining (E). For lipid content analysis cells were incubated with vehicle (DM) plus CA (50 μM), HT (100 μM), LIP (10 μl/ml), or the indicated combination of CA or HT with LIP, or vehicle CT alone, for 72 h (day 7 of culture). mRNA levels of *pparg* (F) and *cebpa* (G) were normalized to the geometric mean of the two reference genes, *ef1a* and *ubiquitin*. For gene expression analyses cells were incubated with vehicle (DM) plus CA (50 μM), HT (100 μM), RGZ (1 μM), LIP (10 μl/ml), or the indicated combination of CA or HT with RGZ or LIP, or vehicle CT alone for 24 h (day 7 of culture). Data are shown as mean ± SEM (n = 3–7 cell cultures). Significant differences (*p* ≤ 0.05) are indicated by different letters, using one-way ANOVA followed by Tukey’s *post hoc* test (B, C, E, F) or the non-parametric Kruskal-Wallis test followed by paired U-Mann Whitney test (G).

CA and HT significantly increased adipocyte viability (S2A Fig) without affecting proliferation (S2B Fig), whether the cells were co-treated with RGZ or not. RGZ alone or in combination with treatments did not affect cell viability or the percentage of PCNA-positive cells (S2 Fig). Quantification of the PPARγ immunofluorescence signal revealed that treating adipocytes with CA or HT in combination with RGZ significantly reduced the enhanced PPARγ protein expression signal produced by RGZ alone (Fig 4A and 4B). A very similar pattern although not showing significant differences was observed concerning PPARγ protein expression by Western blot (Fig 4C and 4D). A lower level of lipid storage was observed when these cells were cultured in the presence of lipid mixture (LIP)+CA and especially in the LIP+HT condition, compared to LIP alone (0.93 ± 0.176 and 0.56 ± 0.047 in LIP+CA and LIP+HT, respectively, *versus* 1.34 ± 0.13 with LIP, *p* ≤ 0.05) (Fig 4E). Finally, qRT-PCR data revealed that LIP significantly increased the expression of *pparg* and *cebpa* transcripts compared to CT, whereas RGZ did not affect these values (Fig 4F and 4G). In addition, treatments with CA or HT slightly down-regulated *pparg* and *cebpa* mRNA levels when combined with LIP, whereas no changes were observed in combination with RGZ (Fig 4F and 4G).

### *In vivo* effects of CA and HT on rainbow trout lipid metabolism

**Fig 5:**
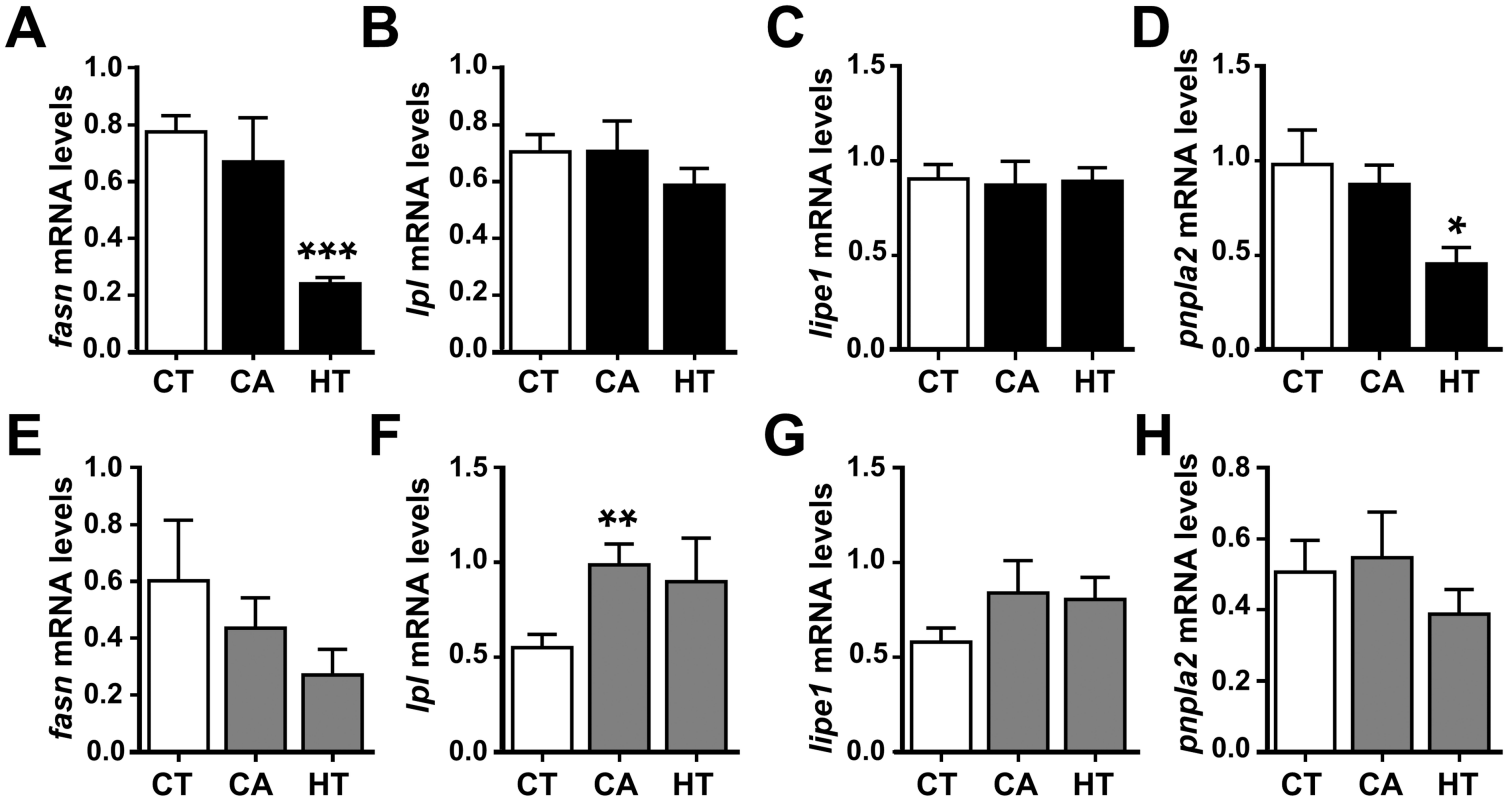
qRT-PCR analysis of selected lipid-metabolism-related gene transcript levels in the dissected perivisceral WAT (A-D) and liver (E-H) of rainbow trout treated with CA and HT or untreated. After one-day fasting, juvenile rainbow trout received intraperitoneal injections of vehicle CT (DMSO in sesame oil 1:3, v/v), vehicle plus CA (10 μg/g body weight), or vehicle plus HT (20 μg/g body weight), and samples were taken after a 24 h exposure period in a fasting state. mRNA levels of *fasn* (A, E), *lpl* (B, F), *lipe1* (C, G) and *pnpla2* (D, H). All mRNA levels were normalized to the geometric mean of the two reference genes, *ef1a* and *actb*. Data are shown as mean ± SEM (n = 7–8 fish per condition). *p ≤ 0.05, **p ≤ 0.01, ***p ≤ 0.0001, compared to CT, using Student's t-test.

**Fig 6:**
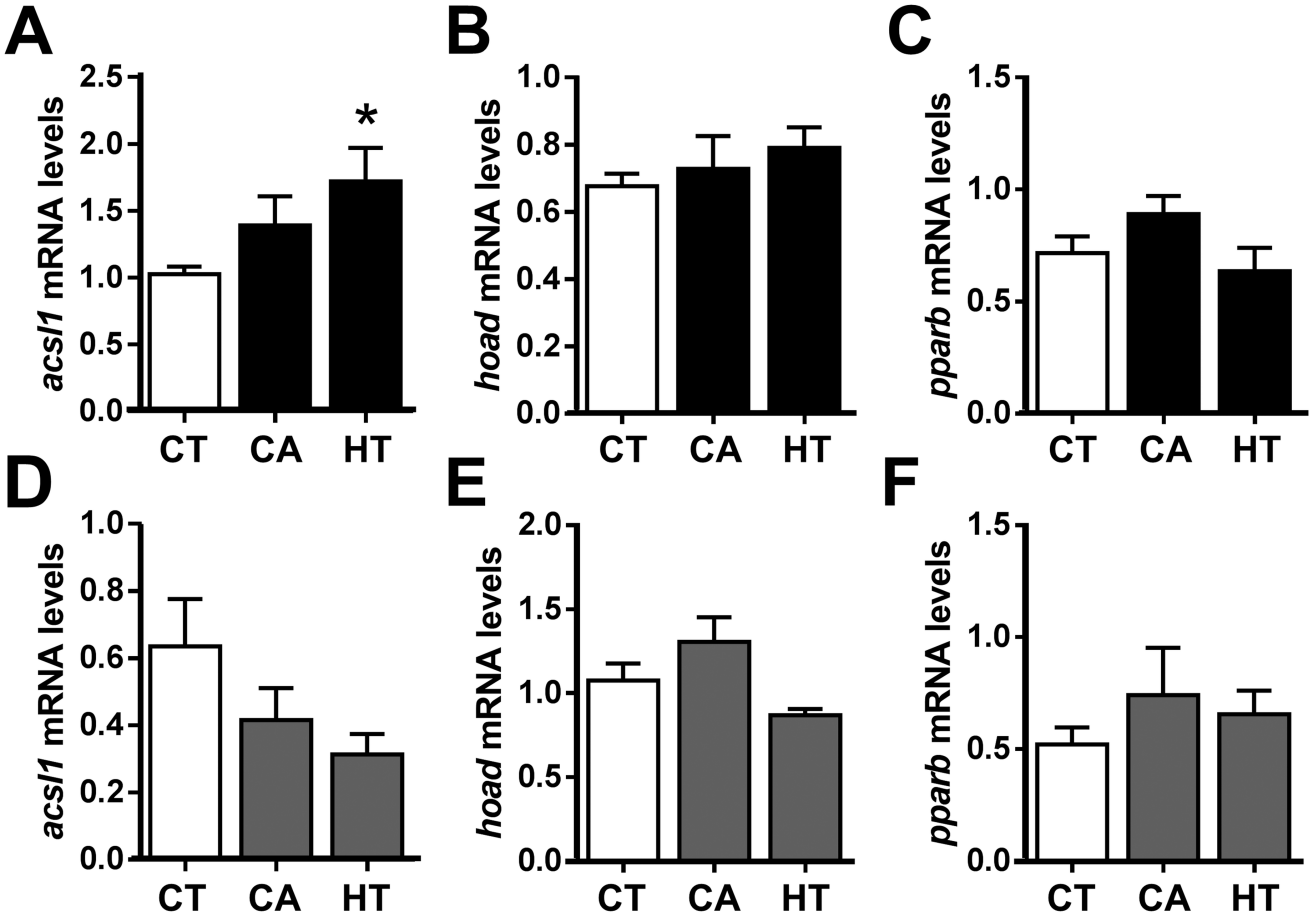
qRT-PCR analysis of selected β-oxidation-related gene transcript levels in the dissected perivisceral WAT (A-C) and liver (D-F) of rainbow trout treated with CA and HT or untreated. After one-day fasting, juvenile rainbow trout received intraperitoneal injections of vehicle CT (DMSO in sesame oil 1:3, v/v), vehicle plus CA (10 μg/g body weight), or vehicle plus HT (20 μg/g body weight), and samples were taken after a 24 h exposure period in a fasting state. mRNA levels of *acsl1* (A, D), *hoad* (B, E) and *pparb* (C, F). All mRNA levels were normalized to the geometric mean of the two reference genes, *ef1a* and *actb*. Data are shown as mean ± SEM (n = 7–8 fish per condition). *p ≤ 0.05, compared to CT, using Student's t-test.

The next step was to clarify the role of CA and HT in an *in vivo* context by focusing on several key adipogenic, lipolitic and β-oxidation markers, as well as the potential cross talk between liver and WAT, using qRT-PCR and plasma parameters analyses. The data revealed that transcriptional levels of *fasn* in both WAT and liver were significantly decreased by HT treatment (Fig 5A and 5E), indicating a reduction in fatty acid synthesis, possibly resulting in a decrease in TAG formation and fat deposits. Moreover, *pnpla2* expression in WAT but not liver decreased significantly following HT administration (Fig 5D and 5H). On the other hand, *lipe1* mRNA levels in both liver and WAT were unaffected by CA or HT treatment (Fig 5C and 5G). Following CA administration, *lpl* mRNA levels were unaffected in WAT, but increased in liver, suggesting a higher TAG lipase activity in this tissue (Fig 5B and 5F). Regarding β-oxidation markers, *acsl1* mRNA levels presented a reverse regulation between tissues, being significantly increased by HT treatment in WAT and showing an opposite pattern but not significant in liver (Fig 6A and 6D). Aside from that, no changes were found in *hoad* (Fig 6B and 6E) nor *pparb* (Fig 6C and 6F) mRNA levels due to treatment in any tissue.

Since CA and HT were shown to modulate the expression of adipogenic, lipolytic and β-oxidation genes that regulate the lipid metabolism in both WAT and liver, it was useful to obtain further information by measuring blood parameters. Analysis of selected biochemical parameters in plasma revealed a slight decrease in TAG levels in fasting rainbow trout injected with CA and a significantly lower concentration with HT compared to CT (Table 1). However, no significant effects upon treatments were observed in plasma glucose nor NEFAs.

**Table 1:** Selected biochemical plasma parameters of rainbow trout intraperitoneally injected with CA or HT.

	CT	CA	HT
TAG (mmol/L)	4.69 ± 0.738	3.53 ± 0.164	2.59 ± 0.478*
Glycerol (DO 540 nm)	0.06 ± 0.0013	0.06 ± 0.0014	0.06 ± 0.0006
Glucose (mmol/L)	8.61 ± 1.077	6.86 ± 0.417	6.83 ± 0.480
NEFAs (mEq/L)	0.96 ± 0.074	1.02 ± 0.086	1.16 ± 0.133

Juvenile rainbow trout kept at 15°C received intraperitoneal injections after a one-day fast with vehicle CT, vehicle plus CA (10 μg/g), or vehicle plus HT (20 μg/g). After 24 h exposure to the compound, blood samples were harvested for further plasma biochemical analyses. The total fasting period was therefore 48 hours before blood sampling. Values are expressed as means ± SEM (n = 7 to 8 fish per condition). TAG, triacylglycerols; NEFAs, non-esterified fatty acids.

*p < 0.05 *versus* control using Student’s t-test.

## Discussion

While mammalian models have largely contributed to improving our understanding of obesity, in recent years, teleost models have also produced excellent findings on human diseases and offer a number of advantages [41]. Despite obvious differences, teleost fish share a significant amount of genetic identity with humans, and some metabolic pathways and organ systems are also remarkably similar [42,43]. Their rapid development ex-utero and the optical semi-transparency of the embryonic and early larval stages, as well as the advances in fish cell culture techniques have made teleost fish popular models in applied and basic research [14,21,41,42]. In mammals, fat deposits in specific regions differ from each other not only by localization but also by their structural and functional properties [44]. Similarly, WAT development in zebrafish is a step-wise process, with differentiated adipocytes first observed in the visceral region, then the subcutaneous area and finally, the head [27]. Moreover, zebrafish fat deposits are also mobilized sequentially, in reverse order, in response to starvation [20]; altogether highlighting the particular suitability of this species to study adiposity dynamics in an *in vivo* scenario.

Natural bioactive phytochemicals have recently attracted interest for their potential health benefits in preventing metabolic diseases, including obesity and insulin resistance [45]. Among these, dietary components of olive oil, red wine and marine algae are particularly interesting, due to their antioxidant properties, which are traditionally linked to longevity and reduced mortality from several diseases [46]. Furthermore, as well as obesogenic molecules, which predispose individuals to metabolic changes leading to weight gain [47], there is growing evidence that some plant compounds can improve overweight by inhibiting adipogenic pathways or impairing lipid accumulation and may, therefore, be classified as anti-obesogenic. Recent studies have reported that some phytochemicals and dietary supplements affect the lipid metabolism in zebrafish. Adding green tea extract to a high-fat diet significantly reduced body fat storage in adults [48,49]. Other studies of very early-stage larvae reported a decrease in lipids after treatment with compounds originating from plants or algae. The protocol consisted of a 15-day exposure to the compound on a high-fat diet background, starting at the first feeding period. For example, curcumin [50–53], indole-3-carbinol [54], baicalein [55], dieckol [56], ellagic acid [52], kaempferol [51], quercetin [56], seapolynol [57] and silibinin [58], have all been reported to inhibit lipid accumulation in these early, post-embryonic developmental stages, when the adipocyte lineage is just starting to be established [20,27]. In our work, we used the ZOT protocol at later larval developmental stages, when the animals had a well-established WAT [30]. *In vivo* Nile red staining of adipocyte lipid droplets and quantitative analysis of whole-mount wide-field fluorescence microscopy signals, before and after a one-day exposure to the selected chemical compounds, were used to monitor the dynamics of WAT mass linked to adipocyte droplet size rather than adipocyte cell number. In addition, this methodology discriminated between the lipid signals associated with WAT and liver and identified the potential modulation of different regional WAT fat deposits in zebrafish larvae.

In this context, the aim of the present study was to investigate the anti-obesogenic properties of three antioxidant compounds of plant origin, selected for their capacity to modulate the lipid metabolism in the 3T3-L1 adipocyte cell line and rodent models [59–64]. ATX is a natural red carotenoid pigment found in a wide variety of organisms, CA is one of the most abundant hydroxycinnamic acids in the human diet, and HT is bioavailable in olive leaves and olive oil. In the present work, CA and HT caused a significant, short-term reduction in zebrafish larval adiposity, but CA exhibited stronger anti-obesogenic effects only in the head region, initiating the reversal of the fat mobilization pattern, as described above, while HT affected both the head and visceral regions. These results indicate that these compounds reduce fat mass by decreasing adipocyte size, but they may have different anti-obesogenic capacities according to the WAT location in the body. As mentioned above, it is known that adipose deposits in different parts of the body have different biological functions and biochemical profiles. Several features, such as adipocyte growth and differentiation, developmental gene expression, susceptibility to apoptosis, inflammatory capacity, and adipokine secretion, vary among deposits, as well as fatty-acid processing and WAT enlargement and loss mechanisms [65]. Nevertheless, further investigation is required to determine how CA and HT target the specific needs of the different deposits. On the other hand, ATX did not modify the adiposity level of zebrafish, even when the different regions were analyzed separately. Although several studies support the hypothesis that ATX supplementation reduces body weight and fat accumulation in mice [66], the ATX action mechanism has been demonstrated to be cell-type dependent [62]. In agreement with our data, one study in humans reported that ATX did not boost fat utilization or fat loss [67].

Adipogenesis is a complex process that typically involves the sequential activation of several transcription factors, such as PPARγ, that regulate the differentiation of pre-adipocytes into mature adipocytes, and ultimately control WAT formation promoting both, lipogenesis and fatty acid uptake. In mammals, it has been demonstrated that CA and HT exhibit a significant potential to act as anti-obesity agents by modulating the PPARγ adipogenesis pathway [64,68]. Therefore, the potential antagonism of PPARγ produced by CA and HT may also disrupt fat accumulation in teleost fish and result in a reduction of WAT mass due to lipid storage impairment. Our results revealed that CA and HT abolished the RGZ-induced obesogenic effect in zebrafish. A similar inhibitory effect has been also reported in zebrafish exposed to specific pharmaceutical antagonists of RGZ [30,69], thus supporting the idea that the compounds used in the present study may antagonize PPARγ signaling.

During the last decade, *in vitro* cell culture models from fish species have been widely used for studies of lipid metabolism and adipocyte functionality [31,70,71]. Nevertheless, despite the increasing use of zebrafish as an obesity model, to our knowledge, a primary adipocyte cell culture from this species has not been developed up to date. In this regard, further investigations using the well-established primary-cultured rainbow trout adipocyte system were performed in order to have a closer look into the potential PPARγ antagonism of CA and HT. The data supported the involvement of PPARγ in the mechanism underlying the anti-obesogenic activity of CA and HT. Concomitant with the counteracting effect downregulating the PPARγ protein expression signal induced by RGZ, CA and especially HT, also reduced the adipocyte lipid content, in agreement with our previous *in vivo* findings in zebrafish. However, qRT-PCR analyses revealed that these two compounds did not modulate the transcript levels of typical key markers of mature adipocytes, i.e. *pparg and cebpa*, when these cells were co-treated with RGZ, and had only a slight effect when they were combined with LIP. These findings suggest that the anti-obesogenic effect of CA and HT may be mediated by post-transcriptional mechanisms.

PPARγ deficiency and/or disruption directly not only affects WAT development and accumulation, but also exerts an impact on the whole body metabolism [72]. The *in vivo* administration experiment in rainbow trout revealed that CA and HT affected the mRNA levels of adipogenic and β-oxidation genes in liver and WAT in different ways. Fat loss is usually caused by the balance of two main processes: enhancement of fat breakdown and inhibition of TAG synthesis and accumulation. This process is orchestrated by the interaction of several tissues, such as WAT, liver and skeletal muscles [73]. The decrease in WAT *fasn* mRNA levels produced by HT treatment was accompanied by a reduction in plasma TAG levels, possibly due to the antagonist effect of this compound on the PPARγ signaling pathway. In agreement with our results, some mammal studies have demonstrated that CA and HT regulate WAT mass by suppressing lipogenic enzyme activity and claimed that PPARγ antagonists can be used to treat hyperlipidemia [74,75]. Interestingly, HT also downregulated *pnpla2* transcript abundance in rainbow trout WAT, which is indicative of decreased lipolysis, leading to a reduction in lipid turnover or compensation for excessive WAT decrease. On the other hand, CA administration increased *lpl* mRNA levels in the liver, also associated with the decreased TAG levels observed in plasma. Concomitant with this decrease, CA and HT also smoothly increased NEFAs levels via TAG hydrolysis. Moreover, a slight decrease was also observed in plasma glucose following treatment, in agreement with mammal studies where CA and HT have been proposed as potential hypoglycemic antidiabetic treatments [76,77]. Within this *in vivo* scenario it is important to notice that CA and HT are antioxidants, with proven effects upon cell viability (S2 Fig), which may have an implication in modulating fatty acid oxidation [78]. In this sense, HT administration upregulated *acsl1* mRNA levels in WAT while slightly decreased its expression in liver, indicating a tissue-specific regulation as previously reported [79]. Even though enhanced *acsl1* expression is traditionally believed to be essential for the synthesis of TAG, several studies in mammals revealed a possible function related to the β-oxidation of fatty acids [80,81], in agreement with our results. Nevertheless, differences were not observed concerning the mRNA levels of *hoad* and *pparb* suggesting that further studies are needed to better understand the potential involvement of CA and HT in WAT β-oxidation.

## Conclusions

The findings reported here provide novel insights into the anti-obesogenic role of CA and HT antioxidants, highlighting their potential involvement in negatively modulating the PPARγ signaling pathway. Further studies using specific inhibitors of these nuclear receptors will help to understand the mechanism of action of these anti-obesogenic compounds. The teleost fish models used have been validated for studying the mode of action of these bioactive compounds on WAT, reinforcing their suitability in pharmacological and biomedical research. This study may also help the investigation of new additives to optimize adiposity in farmed animals.

## Supporting information

S1 Fig ZOT as a tool for studying WAT dynamics in different body parts of zebrafish larva. (A) External features of a representative 8 mm SL larva and images of head, viscera, and tail regions under a fluorescence microscope after Nile red staining, using HQ-FITC-BP filter, with adipocytes stained green. Lateral views, anterior part on the left and dorsal part at the top. (B) Quantitative analysis of adipocyte tissue area in each body region, expressed as a percentage of total adiposity. SL distribution of the animals used was from 7 to 9 mm. Boxplot shows median and percentile adiposity values, n = 11 independent experiments (10 animals per group). Scale bar: 0.5 mm. Abbreviations: ai, anterior intestine; cfa, caudal fin adipocytes; e, eye; pf, pectoral fin; pwat, perivisceral white adipose tissue; sb, swim bladder; swat, subcutaneous white adipose tissue.
(TIF)

S2 Fig CA and HT increased cell viability but did not affect cell proliferation of rainbow trout adipocytes in primary culture. (A) Quantification of cell viability using MTT assay. (B) Cell proliferation determined by immunocytochemistry of PCNA. Cells were incubated with vehicle plus CA (50 μM), HT (100 μM), or RGZ (1 μM), alone or in combination, or vehicle CT alone, for 24 h (day 5 of culture). Data are shown as mean ± SEM (n = 3–4 cell cultures). *p ≤ 0.05, **p ≤ 0.01 compared to CT, using one-way ANOVA test followed by Tukey’s *post hoc* test.
(TIF)

S1 Table Nucleotide sequences of the primers used to evaluate mRNA abundance by qRT-PCR in rainbow trout. (DOC)
