# Supplementary figures and images for: Caffeic acid and hydroxytyrosol have anti-obesogenic properties in zebrafish and rainbow trout models
Source: PLoS One. 2017 Jun 1;12(6):e0178833. doi: 10.1371/journal.pone.0178833 (PMC5453583; doi:10.1371/journal.pone.0178833)

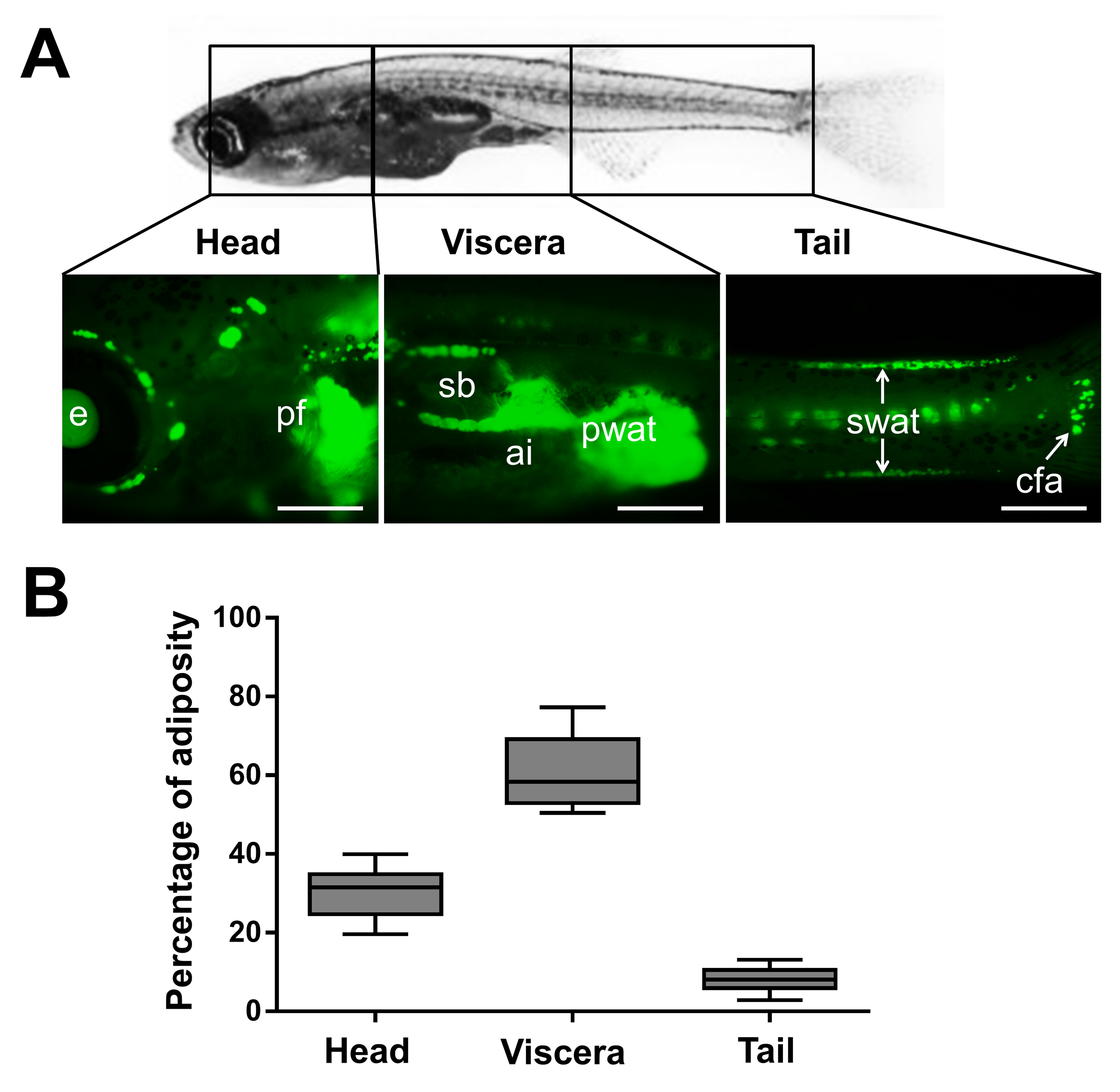

Supplement: S1 Fig — (A) External features of a representative 8 mm SL larva and images of head, viscera, and tail regions under a fluorescence microscope after Nile red staining, using HQ-FITC-BP filter, with adipocytes stained green. Lateral views, anterior part on the left and dorsal part at the top. (B) Quantitative analysis of adipocyte tissue area in each body region, expressed as a percentage of total adiposity. SL distribution of the animals used was from 7 to 9 mm. Boxplot shows median and percentile adiposity values, n = 11 independent experiments (10 animals per group). Scale bar: 0.5 mm. Abbreviations: ai, anterior intestine; cfa, caudal fin adipocytes; e, eye; pf, pectoral fin; pwat, perivisceral white adipose tissue; sb, swim bladder; swat, subcutaneous white adipose tissue. (TIF) [file pone.0178833.s001.tif]

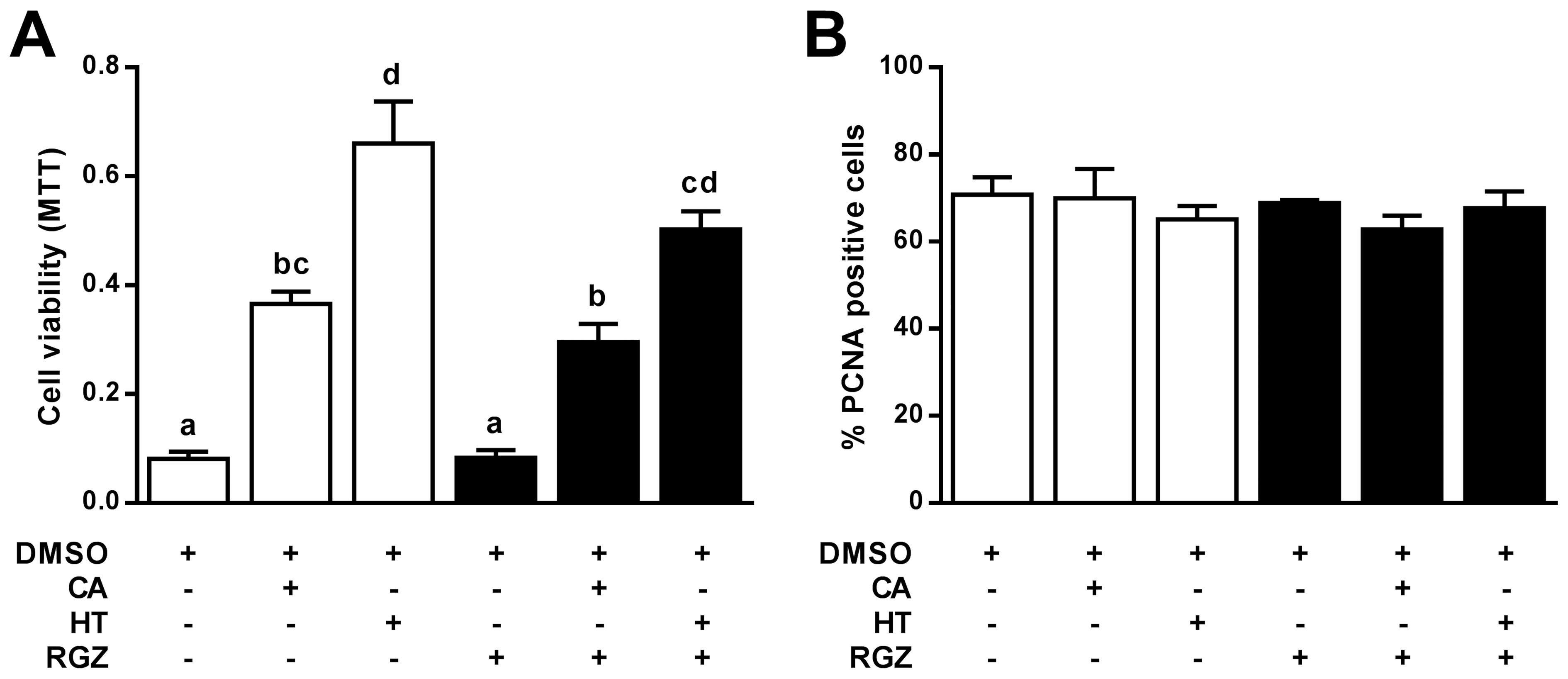

Supplement: S2 Fig — (A) Quantification of cell viability using MTT assay. (B) Cell proliferation determined by immunocytochemistry of PCNA. Cells were incubated with vehicle plus CA (50 μM), HT (100 μM), or RGZ (1 μM), alone or in combination, or vehicle CT alone, for 24 h (day 5 of culture). Data are shown as mean ± SEM (n = 3–4 cell cultures). *p ≤ 0.05, **p ≤ 0.01 compared to CT, using one-way ANOVA test followed by Tukey’s post hoc test. (TIF) [file pone.0178833.s002.tif]
