# Supplementary material for: Caffeic acid and hydroxytyrosol have anti-obesogenic properties in zebrafish and rainbow trout models
Source: PLoS One. 2017 Jun 1;12(6):e0178833. doi: 10.1371/journal.pone.0178833 (PMC5453583; doi:10.1371/journal.pone.0178833)
Supplement: S1 Table — (DOC) [file pone.0178833.s003.doc]

**Table S1.** Nucleotide sequences of the primers used to evaluate mRNA abundance by qRT-PCR in rainbow trout

| **Gene** | | **Primer sequences (5’–3’)** | **Tm (ºC)** | | **Amplicon**  **size (bp)** | | **Database** | **Accession**  **number** | |
| --- | --- | --- | --- | --- | --- | --- | --- | --- | --- |
| *acsl1* | **F**: TGCAATCTAGCAAGGTTCCTTTTG  **R**: TCCAAGCAGAAACCCAGTACAGAA | | | 60 | 137 | Sigenae | | | CR363150.p.om.8 |
| *actb* | **F**: ATCCTGACGGAGCGCGGTTACAGC  **R**: TGCCCATCTCCTGCTCAAAGTCCA | | | 61 | 112 | Genbank | | | AJ438158 |
| *cebpa* | **F**: TGTGGCGATAAAGCAAGAGC  **R**: CTGGTGGGAATGGTGGTAGG | | | 57 | 79 | Genbank | | | DQ423469.1 |
| *ef1α* | **F**: TCCTCTTGGTCGTTTCGCTG  **R**: ACCCGAGGGACATCCTGTG | | | 58 | 159 | Genbank | | | NM_001124339.1 |
| *fasn* | **F**: GAGACCTAGTGGAGGCTGTC  **R**: TCTTGTTGATGGTGAGCTGT | | | 54 | 186 | Sigenae | | | tcaa0001c.m.06_5.1.om.4 |
| *hoad* | **F**: GGACAAAGTGGCACCAGCAC  **R**: GGGACGGGGTTGAAGAAGTG | | | 59 | 145 | Sigenae | | | tcad0001a.i.15 3.1.om |
| *lipe1* | **F**: AGGGTCATGGTCATCGTCTC  **R**: CTTGACGGAGGGACAGCTAC | | | 58 | 175 | Genbank | | | NM_001197209.1 |
| *lpl* | **F**: TAATTGGCTGCAGAAAACAC  **R**: CGTCAGCAAACTCAAAGGT | | | 59 | 164 | Genbank | | | AJ224693 |
| *pnpla2* | **F**: CGTGTCCGAGTTCAAGTC  **R**: GGAGAGATGCTGATGGTG | | | 56 | 174 | Genbank | | | BX318925.2 |
| *pparb* | **F**: CTGGAGCTGGATGACAGTGA  **R**: GTCAGCCATCTTGTTGAGCA | | | 59 | 195 | Genbank | | | AY356399.1 |
| *pparg* | **F**: GCCAGTACTGTCGCTTTCAG  **R**: TCCATAAACTCAGCCAGCAG | | | 60 | 171 | Genbank | | | NM_001197212.1 |
| *ubiquitin* | **F**: ACAACATCCAGAAAGAGTCCA  **R**: AGGCGAGCGTAGCACTTG | | | 58 | 133 | Genbank | | | NM_001124194.1 |

F: forward; R: reverse; Tm: melting temperature; bp: base pairs.
